# The NMDA receptor partial agonist d-cycloserine does not enhance motor learning

**DOI:** 10.1177/0269881116658988

**Published:** 2016-07-19

**Authors:** Jan Günthner, Jacqueline Scholl, Elisa Favaron, Catherine J Harmer, Heidi Johansen-Berg, Andrea Reinecke

**Affiliations:** 1 Department of Psychiatry, University of Oxford, Oxford, UK; 2 Department of Addictive Behaviour and Addiction Medicine, Central Institute of Mental Health (CIMH), University of Heidelberg, Medical Faculty Mannheim, Mannheim, Germany; 3 Department of Experimental Psychology, University of Oxford, Oxford, UK; 4 Oxford Centre for Functional MRI of the Brain, University of Oxford, Oxford, UK

**Keywords:** NMDA, motor learning, d-cycloserine

## Abstract

**Rationale:**

There has recently been increasing interest in pharmacological manipulations that could potentially enhance exposure-based cognitive behaviour therapy for anxiety disorders. One such medication is the partial NMDA agonist d-cycloserine. It has been suggested that d-cycloserine enhances cognitive behaviour therapy by making learning faster. While animal studies have supported this view of the drug accelerating learning, evidence in human studies has been mixed. We therefore designed an experiment to measure the effects of d-cycloserine on human motor learning.

**Methods:**

Fifty-four healthy human volunteers were randomly assigned to a single dose of 250mg d-cycloserine versus placebo in a double-blind design. They then performed a motor sequence learning task.

**Results:**

D-cycloserine did not increase the speed of motor learning or the overall amount learnt. However, we noted that participants on d-cycloserine tended to respond more carefully (shifting towards slower, but more correct responses).

**Conclusion:**

The results suggest that d-cycloserine does not exert beneficial effects on psychological treatments via mechanisms involved in motor learning. Further studies are needed to clarify the influence on other cognitive mechanisms.

## Introduction

The N-methyl-D-aspartate (NMDA) receptor partial agonist d-cycloserine (DCS) has been found to enhance diverse kinds of learning in animal studies, including fear extinction (Walker et al., 2002), cocaine cue extinction (Torregrossa et al., 2010) and reward learning (Golden and Houpt, 2007; Portero-Tresserra et al., 2013).

In humans, results have so far been mixed, with some studies reporting that DCS could enhance motor learning (Kuriyama et al., 2011), fear conditioning (Kalisch et al., 2009) or incremental learning (Forsyth et al., 2015), but others failing to find an effect on motor learning (Cherry et al., 2014; Feld et al., 2013; Kuo et al., 2008), fear extinction (Guastella et al., 2007; Klumpers et al., 2012) or reward learning (Scholl et al., 2014).

Assessing the effect of DCS on learning further is important with respect to its possible clinical relevance: DCS has been proposed to enhance psychotherapy (Hofmann et al., 2013, 2015), in particular the treatment of anxiety disorders, based on cognitive behavioural therapy. (Bontempo et al., 2012; Norberg et al., 2008; Rodrigues et al., 2014; but see also Ori et al., 2015). It has been suggested that enhancement through DCS is based on its effect on synaptic plasticity (in which NMDA receptors are crucially involved), which has been proposed as the molecular level correlate of behavioural learning (Cain, 1997).

In this study, we tested the impact of DCS on motor learning further, in particularly the effects of a higher dose than tested previously (Feld et al., 2013; Kuo et al., 2008; Kuriyama et al., 2011) . We tested human participants on a motor sequence learning task after administering a single dose of 250mg of DCS or placebo. We found that DCS did not affect motor learning or consolidation of motor learning.

DCS might exert its clinical effects via learning-independent cognitive mechanisms in which NMDA receptors play a role, such as evidence integration or decision-making (Floresco et al., 2008; Scholl et al., 2014; Shen et al., 2010; Standage and Pare, 2011; Standage et al., 2013). In an exploratory analysis of non-learning dependent effects we found that DCS led participants to shift their balance in how quickly and accurately they responded towards more careful responding compared with placebo.

## Methods

### Participants

Fifty-four healthy volunteers (28 females) aged 18 to 30 years (mean age 22) participated in the study after having given written informed consent (see Table S1 in the Supplementary Material online for sample characteristics and inclusion criteria). The study was approved by the local ethics committee.

### General procedure

Participants were randomly allocated to receive DCS (250mg) or matching placebo capsules in a double-blind design. Dosing of 250 mg was chosen in accordance with previous studies (Klumpers et al., 2012; Onur et al., 2010) as an intermediate dose within a range of 50mg to 500mg in which cognitive effects of DCS have been tested. Participants fasted for 2h before the testing visit to prevent nutrition dependent influences on drug intake. Testing took place from 10:00 h onwards. Participants were tested on a motor sequence learning task 3h after drug administration. According to product information (King’s Pharmaceutical), plasma peak levels are reached within 3–4h; other studies (Patel et al., 2011; van Berckel et al., 1997, 1998) found that peak levels are reached within 1 h. However, given DCS’ half-life of 8–12 h (product information) or 15 h (Patel et al., 2011), plasma levels would have been close to peak levels during testing, given either time-to-peak information. Consolidation effects were measured at 2h and 24h after the initial motor learning.

### Motor task description

Participants performed a visually cued reaction time task, as previously described in Stagg et al. (2011), responding as quickly and as accurately as possible to a star symbol (cue) appearing in fast succession in one of four possible side-by-side positions (positions 1, 2, 3 and 4) on the screen (Figure S1 in the Supplementary Material online). With the four fingers of their right hand, participants used four buttons of a standard computer keyboard to react in accordance with a star’s position on the screen. In each of 15 sequence learning blocks, three repeats of a fixed 10-digit sequence were presented. In the sequence, there were no directly adjacent repeats of the four positions, that is, participants were not cued to press the same button more than once successively. The cues were presented at a rate of one cue every 1.03s. The presentation of the cues was not dependent on participants’ responses, the sequence continued independent of whether participants pressed the correct button, a wrong button or no button. Participants were instructed to respond as quickly and accurately as possible. For practice, participants initially performed a 30-trial ‘training block’ consisting of visual cue positions in random order. As a manipulation check, another 30-trial ‘random block’ was presented after 13 sequence learning blocks, to confirm that improvements in reaction time throughout the series of 15 sequence blocks occurred due to learning a specific sequence rather than generic motor skill improvement. For clarity, throughout the paper blocks are labelled from 1 to 17 with blocks 1 and 15 being the random sequence blocks.

Previous research (Kuriyama et al., 2011) has suggested that effects of DCS on motor learning might only be seen after a consolidation phase. We therefore additionally tested participants on a brief version of the motor task, including three sequence blocks, two and 24 hours after the main motor task.

### Data analysis

All analyses were performed in Matlab and SPSS. For violations of sphericity in analysis of variance (ANOVA) analyses, Greenhouse–Geisser correction was used.

### Pre-processing

We recorded participants’ reaction time and choice accuracy for each cue shown. Errors consisted of omitted responses, as well as pressing of an incorrect button. Reaction times for error trials were discarded. As participants started to develop anticipatory responses with learning (Figure S2B and Supplementary Results online), we included reaction times of up to 300ms before the onset of the cue on the screen (negative reaction times) in the analysis. To ensure that these early reaction times were really responses to the subsequent cue and not late responses to the previous one, we only included them as responses to the subsequent cue if the response was the correct button with respect to the subsequent cue (a control analysis revealed that in this early time window 92% of responses were correct with respect to the subsequent cue). We transformed reaction times by the natural logarithm. As log transformation is only possible on positive values, we added a fixed 300ms to each reaction time value (i.e. the earliest anticipatory responses were coded as at time > 0ms).

### Assessing learning

In line with previous research (Stagg et al., 2011) learning in this task was measured as an improvement in reaction time across the task. To assess most generally whether learning occurred, we performed an ANOVA for reaction time over all learning blocks (i.e. blocks 2 to 14 and 16 to 17) including group (two levels) and block (15 levels) as factors.

### Balancing how fast and correctly to respond

Previous studies suggested that NMDA receptors not only play a role in learning, but are also involved in other functions, such as decision-making. In the present task, participants did not have to make any decisions per se, such as choosing between different options. Nevertheless, as they had been instructed to respond ‘as fast and accurately as possible’, they had to decide how to strike a balance between these two constraints (we note that over all blocks, reaction time and accuracy correlated positively (*r*_τ_ = 0.20, *p*=0.032), supporting the idea of a trade-off). For example they could respond faster by pressing a key before the appearance of the visual cue, based on their learnt expectation of the next target in the 10-digit sequence. Or instead they could maximize their accuracy by only responding after the visual cue appeared on the screen. In an exploratory analysis, we wanted to examine whether DCS affected how this balance was set. As a simple measure of this balance, we multiplied the log-transformed (and range normalized, range set to between 1 and 10) reaction times (for each block) by the (range normalized) accuracy (for each block), called here ‘Balance score’. High values then indicated that participants responded correctly and slowly while low values indicated less accurate but faster responding. We compared how the groups differed throughout learning using ANOVA and *t*-tests.

### Assessing consolidation

Additional data was collected on a shorter three-block version of the same sequence task at 2h and 24h after the initial learning to assess effects of DCS on motor learning consolidation. As DCS has a long half-life and we used a high dose of DCS compared with previous studies, we expected participants to still have relatively high DCS levels after the end of testing, which could improve consolidation. Reaction time and Balance score group differences were again assessed using ANOVAs.

## Results

DCS did not affect participants’ mood or arousal (Table S2 online).

### Initial learning performance

**Figure 1.**
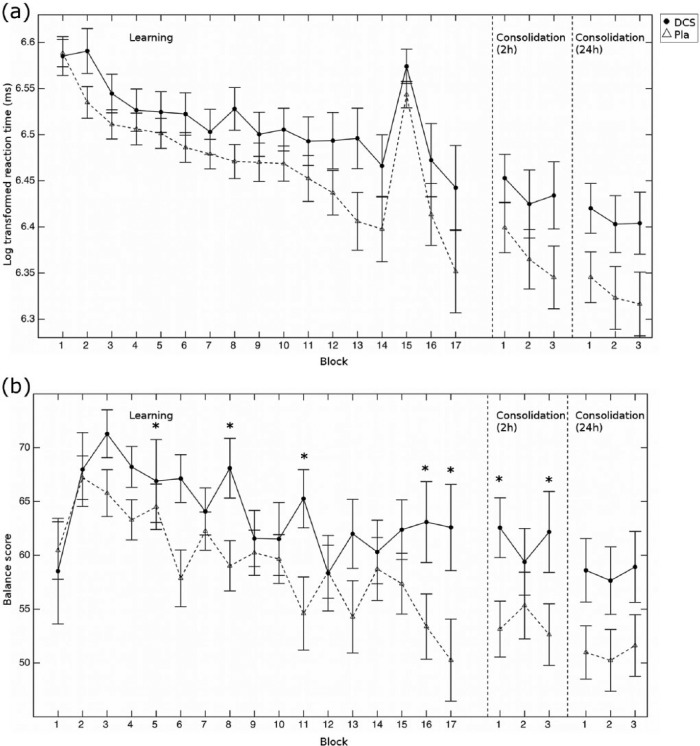
Behavioural results. (a) Plot of the log-transformed reaction times across the learning and the two consolidation phases for the d-cycloserine (circles) and the placebo (triangles, dashed line) groups. The initial learning blocks were the 15 sequence learning blocks (blocks 2 to 14, 16 and 17) while participants performed a random sequence in blocks 1 and 15. Each consolidation phase consisted of three sequence blocks. There were no group differences in either the initial learning or the consolidation (all *p*>0.9). (b) Balance scores (i.e. log-transformed reaction times multiplied by accuracy) across the learning task and consolidation. Error bars show the standard error of the mean, *p<0.055.

We assessed whether DCS could enhance motor learning. We found (Figure 1(a), ‘Learning’) that participants learnt the task well, as indicated by an improvement in log reaction times with learning across all 15 learning blocks (ANOVA, effect of block (15): *F*(2.2,112.9)=14.9, *p*<0.001). While participants had reaction times of 412±11ms in the first learning block, they had speeded up to 322±17ms in the last learning block. However, this improvement in reaction time across all 15 learning blocks did not differ between the groups (ANOVA, block (15) × group (2) interaction: *F*(2.2,112.9)=1.1, *p*=0.34), nor was there a difference in the average reaction time between groups (ANOVA, main effect of group: *F*(1,52)=2.10 *p*=0.15). Similarly, DCS did not affect either just the early speed of learning or the total amount of learning (see Supplementary Methods and Results and Figure S2 online). As accuracy was already very high in the first learning block, we did not analyse accuracy improvements as a measure of learning (Figure S2C).

### Balancing how fast and correctly to respond

In an exploratory analysis we examined whether DCS had effects beyond learning. Specifically we computed a ‘Balance score’ of response speed and correctness. High values then indicated slow but accurate responding while low scores indicated fast but inaccurate responding. We found that the groups significantly differed in their Balance score (Figure 1(b), ANOVA, group × block interaction: *F*(6.9, 359.3)=2.3, *p*=0.029). While participants in the placebo group shifted their responding towards faster but less accurate responding over the course of learning, this was not true in the DCS group (Balance score differences between the last and first learning block (i.e. block 17 minus block 2), group difference: *t*(52)= −2.24, *p*=0.023; after controlling for block 2 as a covariate: *F*(1,53)=5.53, *p*=0.023). Specifically, while the two groups did not differ in the first learning block (*t*(52)= −0.2, *p*=0.84), they differed in the last two blocks of learning (block 16: *t*(52)= −2.0, *p*=0.052, Cohen’s *d*= −0.56; block 17: *t*(52)= −2.1, *p*=0.040, Cohen’s *d*= −0.61). Similarly, when instead of using the Balance score, we performed an ANOVA with type (range normalized log reaction time (logRT) or accuracy) × group, we found that in block 17, there was a group difference (*F*(1,52)=4.50, *p*=0.039). This was in the absence of a group difference on logRT (*t*(52)= −1.33, *p*=0.19) or accuracy (*t*(52)= −1.78, *p*=0.082) alone. These results suggested that our data provides some preliminary evidence that DCS may shift the balance in responding, shifting it towards slower but more accurate responding.

### Consolidation

There was no effect of DCS on consolidation of learning: we found that neither after 2h, nor after 24h (Figure 1(a) ‘Consolidation (2h)/(24h)’) did participants differ in their reaction times (2h: ANOVA, main effect of group: *F*(1,52)=2.1, *p*=0.15, block × group interaction: *F*(2,104)=1.56, *p*=0.22; 24h: ANOVA, main effect of group: *F*(1,52)=2.9, *p*=0.09, block × group interaction: *F*(1.8,92.0)=2.7, *p*=0.74). Similarly, they did not differ in how much their reaction times changed either between the last three blocks of the initial learning and the 2h consolidation (ANOVA, session × group interaction: *F*(1,104)=0.06, *p*=0.8) or between the 2h and the 24h consolidation measure (ANOVA, session × group interaction: *F*(1,104)=0.67, *p*=0.42).

We also looked at the Balance scores in the consolidation blocks. We hypothesized that at least for the 2h consolidation, the groups should still differ because of DCS’s long half-life of 8–15h, which would mean that DCS levels should still be high during this first consolidation phase. We found marginal evidence for this: in the consolidation test after 2h, the DCS group showed a trend for higher Balance score (Figure 1(b) ‘Consolidation (2h)/(24h)’, ANOVA, main effect of group: *F*(1,52)=3.4, *p*=0.071). This group difference was similar at the 24h consolidation test (ANOVA, main effect of group: *F*(1,52)=3.5, *p*=0.068); the difference between the 2h and the 24h consolidation test was not significant (ANOVA, session × group interaction: *F*(1,104)=0.01, *p*=0.91; main effect of group across both sessions: *F*(1,52)=3.65, *p*=0.062).

## Discussion

### DCS does not enhance motor sequence learning

In this study we tested whether a single dose (250mg) of DCS could enhance motor learning. In the task, participants had to learn to tap out a simple motor sequence, following visual cues on a computer screen. While participants showed improvements in reaction times with learning, this was not affected by DCS. There was also no drug effect on the consolidation of motor learning after 2h or after 24h. This result is in agreement with several other studies that have failed to find DCS (at doses of 100mg, 175mg or 250mg) to enhance motor learning (Cherry et al., 2014; Feld et al., 2013; Kuo et al., 2008). However, we note that Kuriyama et al. (2011) found that 100mg of DCS could enhance motor sequence performance after consolidation learning. These discrepancies could be due to the specifics of the task or difference in drug dose or timing of DCS administration (Kuriyama et al. administered DCS 1.5h before the task). Effects of DCS are strongly dose-dependent (Walker et al., 2002) and discrepancies between human and animal studies might be due to the fact that human studies tended to use a lower dose (about three times smaller than those used in animal studies). Lastly, it is also possible that the true effects of DCS on motor sequence learning have a small effect size and therefore only reach significance in some studies with the level of statistical power commonly employed.

### DCS may shift the balance between responding quickly and accurately

It has been suggested that DCS could influence decision-making or integration of evidence for decision-making, based on neural network simulation studies (Standage and Pare, 2011; Standage et al., 2013; Wang, 2002) and empirical work implicating NMDA receptors in this process (Floresco et al., 2008; Meuwese et al., 2013; Scholl et al., 2014; Self et al., 2012; Shen et al., 2010). We therefore performed an exploratory analysis of whether DCS shifted the balance of how quickly and how accurately participants responded. We found that DCS shifted participants’ response towards more careful, namely slower but more correct, responding. However, the effect was not strong, only reaching significance in the late phase of the initial learning, possibly due to low task sensitivity. Our measure of ‘balance in responding’ might be related to the speed/accuracy trade-off that has been measured in decision-making tasks (Britten et al., 1992) and that has been proposed to be implemented by changes to NMDA receptor conductance (Standage et al., 2013). We do stress, however, that our main objective was to test learning effects of DCS, and the motor learning task we used was not a specifically designed task to measure a speed/accuracy trade-off. Our study can therefore be seen as a first hint that DCS may affect the speed/accuracy trade-off, but future studies, using specifically designed tasks (e.g. Bogacz et al., 2010), will be needed to clarify this and to see whether this effect of DCS may be related to its clinical properties. For example, DCS could help patients with anxiety disorders, who might tend to decide too quickly that stimuli are threatening, to first reflect on them and then decide more accurately.

## Conclusion

We found that a single dose of 250mg of DCS did not affect motor sequence learning or consolidation in humans. An exploratory analysis revealed a possible influence of DCS on participants’ response strategy, leading to slower but more accurate responding.

## Supplementary Material

Supplementary material

## References

[bibr1-0269881116658988] Bogacz R Wagenmakers EJ Forstmann BU (2010) The neural basis of the speed-accuracy tradeoff. Trends Neurosci 33: 10–6. 19819033 10.1016/j.tins.2009.09.002

[bibr2-0269881116658988] Bontempo A Panza KE Bloch MH (2012) D-cycloserine augmentation of behavioral therapy for the treatment of anxiety disorders: A meta-analysis. J Clin Psychiatry 73: 533–537. 22579153 10.4088/JCP.11r07356 PMC3625928

[bibr3-0269881116658988] Britten KH Shadlen MN Newsome WT (1992) The analysis of visual motion: A comparison of neuronal and psychophysical performance. J Neurosci 12: 4745–4765. 1464765 10.1523/JNEUROSCI.12-12-04745.1992 PMC6575768

[bibr4-0269881116658988] Cain DP (1997) LTP, NMDA, genes and learning. Curr Opin Neurobiol 7: 235–242. 9142751 10.1016/s0959-4388(97)80012-8

[bibr5-0269881116658988] Cherry KM Lenze EJ Lang CE (2014) Combining d-cycloserine with motor training does not result in improved general motor learning in neurologically intact people or in people with stroke. J Neurophysiol 111: 2516–2524. 24671538 10.1152/jn.00882.2013 PMC4044442

[bibr6-0269881116658988] Feld GB Lange T Gais S (2013) Sleep-dependent declarative memory consolidation–unaffected after blocking NMDA or AMPA receptors but enhanced by NMDA coagonist D-cycloserine. Neuropsychopharmacology 38: 2688–2697. 23887151 10.1038/npp.2013.179 PMC3828540

[bibr7-0269881116658988] Floresco SB Tse MT Ghods-Sharifi S (2008) Dopaminergic and glutamatergic regulation of effort- and delay-based decision making. Neuropsychopharmacology 33: 1966–1979. 17805307 10.1038/sj.npp.1301565

[bibr8-0269881116658988] Forsyth JK Bachman P Mathalon DH (2015) Augmenting NMDA receptor signaling boosts experience-dependent neuroplasticity in the adult human brain. Proc Nat Acad Sci 112: 15331–15336. 26621715 10.1073/pnas.1509262112 PMC4687562

[bibr9-0269881116658988] Golden GJ Houpt TA (2007) NMDA receptor in conditioned flavor-taste preference learning: Blockade by MK-801 and enhancement by D-cycloserine. Pharmacol Biochem Behav 86: 587–596. 17350084 10.1016/j.pbb.2007.02.004 PMC2570030

[bibr10-0269881116658988] Guastella AJ Lovibond PF Dadds MR (2007) A randomized controlled trial of the effect of D-cycloserine on extinction and fear conditioning in humans. Behav Res Ther 45: 663–672. 16962066 10.1016/j.brat.2006.07.005

[bibr11-0269881116658988] Hofmann SG Otto MW Pollack MH (2015) D-cycloserine augmentation of cognitive behavioral therapy for anxiety disorders: an update. Curr Psychiatry Rep 17: 532. 25413638 10.1007/s11920-014-0532-2

[bibr12-0269881116658988] Hofmann SG Wu JQ Boettcher H (2013) D-cycloserine as an augmentation strategy for cognitive behavioral therapy of anxiety disorders. Biol Mood Anxiety Disord 3: 1–10. 23768232 10.1186/2045-5380-3-11 PMC3686620

[bibr13-0269881116658988] Kalisch R Holt B Petrovic P (2009) The NMDA agonist D-cycloserine facilitates fear memory consolidation in humans. Cereb Cortex 19: 187–196. 18477687 10.1093/cercor/bhn076 PMC2638747

[bibr14-0269881116658988] Klumpers F Denys D Kenemans JL (2012) Testing the effects of Delta9-THC and D-cycloserine on extinction of conditioned fear in humans. J Psychopharmacol 26: 471–478. 22351380 10.1177/0269881111431624 PMC3454470

[bibr15-0269881116658988] Kuo MF Unger M Liebetanz D (2008) Limited impact of homeostatic plasticity on motor learning in humans. Neuropsychologia 46: 2122–2128. 18394661 10.1016/j.neuropsychologia.2008.02.023

[bibr16-0269881116658988] Kuriyama K Honma M Koyama S (2011) D-cycloserine facilitates procedural learning but not declarative learning in healthy humans: A randomized controlled trial of the effect of D-cycloserine and valproic acid on overnight properties in the performance of non-emotional memory tasks. Neurobiol Learn Mem 95: 505–509. 21402164 10.1016/j.nlm.2011.02.017

[bibr17-0269881116658988] Meuwese JD Van Loon AM Scholte HS (2013) NMDA receptor antagonist ketamine impairs feature integration in visual perception. PLoS One 8: e79326. 24223927 10.1371/journal.pone.0079326 PMC3815103

[bibr18-0269881116658988] Norberg MM Krystal JH Tolin DF (2008) A meta-analysis of D-cycloserine and the facilitation of fear extinction and exposure therapy. Biol Psychiatry 63: 1118–1126. 18313643 10.1016/j.biopsych.2008.01.012

[bibr19-0269881116658988] Onur OA Schlaepfer TE Kukolja J (2010) The N-methyl-D-aspartate receptor co-agonist D-cycloserine facilitates declarative learning and hippocampal activity in humans. Biol Psychiatry 67: 1205–1211. 20303474 10.1016/j.biopsych.2010.01.022

[bibr20-0269881116658988] Ori R Amos T Bergman H (2015) Augmentation of cognitive and behavioural therapies (CBT) with d-cycloserine for anxiety and related disorders. Cochrane Database System Rev 5: CD007803–CD007803. 25957940 10.1002/14651858.CD007803.pub2 PMC8939046

[bibr21-0269881116658988] Patel DS Sharma N Patel MC (2011) Development and validation of a selective and sensitive LC-MS/MS method for determination of cycloserine in human plasma: Application to bioequivalence study. J Chromatogr B Analyt Technol Biomed Life Sci 879: 2265–2273. 10.1016/j.jchromb.2011.06.011 21727043

[bibr22-0269881116658988] Portero-Tresserra M Marti-Nicolovius M Guillazo-Blanch G (2013) D-cycloserine in the basolateral amygdala prevents extinction and enhances reconsolidation of odor-reward associative learning in rats. Neurobiol Learn Mem 100: 1–11. 23200640 10.1016/j.nlm.2012.11.003

[bibr23-0269881116658988] Rodrigues H Figueira I Lopes A (2014) Does D-cycloserine enhance exposure therapy for anxiety disorders in humans? A meta-analysis. PLoS One 9: e93519. 24991926 10.1371/journal.pone.0093519 PMC4081005

[bibr24-0269881116658988] Scholl J Gunthner J Kolling N (2014) A role beyond learning for NMDA receptors in reward-based decision-making-a pharmacological study using d-cycloserine. Neuropsychopharmacology 39: 2900–2909. 24924800 10.1038/npp.2014.144 PMC4200501

[bibr25-0269881116658988] Self MW Kooijmans RN Supèr H (2012) Different glutamate receptors convey feedforward and recurrent processing in macaque V1. Proc Natl Acad Sci U S A 109: 11031–11036. 22615394 10.1073/pnas.1119527109 PMC3390882

[bibr26-0269881116658988] Shen K Kalwarowsky S Clarence W (2010) Beneficial effects of the NMDA antagonist ketamine on decision processes in visual search. J Neurosci 30: 9947–9953. 20660277 10.1523/JNEUROSCI.6317-09.2010 PMC6632808

[bibr27-0269881116658988] Stagg CJ Bachtiar V Johansen-Berg H (2011) The role of GABA in human motor learning. Curr Biol 21: 480–484. 21376596 10.1016/j.cub.2011.01.069 PMC3063350

[bibr28-0269881116658988] Standage D Pare M (2011) Persistent storage capability impairs decision making in a biophysical network model. Neural Netw 24: 1062–1073. 21658905 10.1016/j.neunet.2011.05.004

[bibr29-0269881116658988] Standage D You H Wang DH (2013) Trading speed and accuracy by coding time: A coupled-circuit cortical model. PLoS Comput Biol 9: e1003021. 23592967 10.1371/journal.pcbi.1003021 PMC3617027

[bibr30-0269881116658988] Torregrossa MM Sanchez H Taylor JR (2010) D-cycloserine reduces the context specificity of pavlovian extinction of cocaine cues through actions in the nucleus accumbens. J Neurosci 30: 10526–10533. 20685995 10.1523/JNEUROSCI.2523-10.2010 PMC2918879

[bibr31-0269881116658988] Van Berckel BNM Lipsch C Gispen-De Wied C (1998) The partial NMDA agonist D-cycloserine stimulates LH secretion in healthy volunteers. Psychopharmacology 138: 190–197. 9718289 10.1007/s002130050662

[bibr32-0269881116658988] Van Berckel BNM Lipsch C Timp S (1997) Behavioral and neuroendocrine effects of the partial NMDA agonist dcycloserine in healthy subjects. Neuropsychopharmacology 16: 317–324. 9109102 10.1016/S0893-133X(96)00196-0

[bibr33-0269881116658988] Walker DL Ressler KJ Lu K-T (2002) Facilitation of conditioned fear extinction by systemic administration or intra-amygdala infusions of D-cycloserine as assessed with fear-potentiated startle in rats. J Neurosci 22: 2343–2351. 11896173 10.1523/JNEUROSCI.22-06-02343.2002 PMC6758267

[bibr34-0269881116658988] Wang X-J (2002) Probabilistic decision making by slow reverberation in cortical circuits. Neuron 36: 955–968. 12467598 10.1016/s0896-6273(02)01092-9

