# Supplementary material for: The NMDA receptor partial agonist d-cycloserine does not enhance motor learning
Source: J Psychopharmacol. 2016 Jul 19;30(10):994–9. doi: 10.1177/0269881116658988 (PMC5066480; doi:10.1177/0269881116658988)
Supplement: Supplementary material [file JOP658988_supplementary_material.pdf]

The NMDA receptor partial agonist d-cycloserine does not enhance motor learning

Jan Günthner[1,2*], Jacqueline Scholl[1,3*], Elisa Favaron[1], Catherine J. Harmer[1], Heidi Johansen-Berg[4], Andrea Reinecke[1]

Supplementary materials

Supplementary Figures

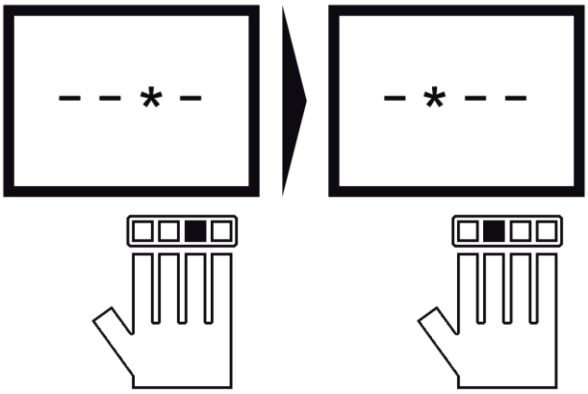

Figure S1. Task design. On each trial, participants saw a cue (star symbol) in one of four positions on the screen. In the example shown here, on the first trial the cue is present in position three, on the second trial in position two. In response to the cue, participants then had to press the corresponding keyboard button with their right hand. The star symbol disappeared from the screen in between cue presentations.

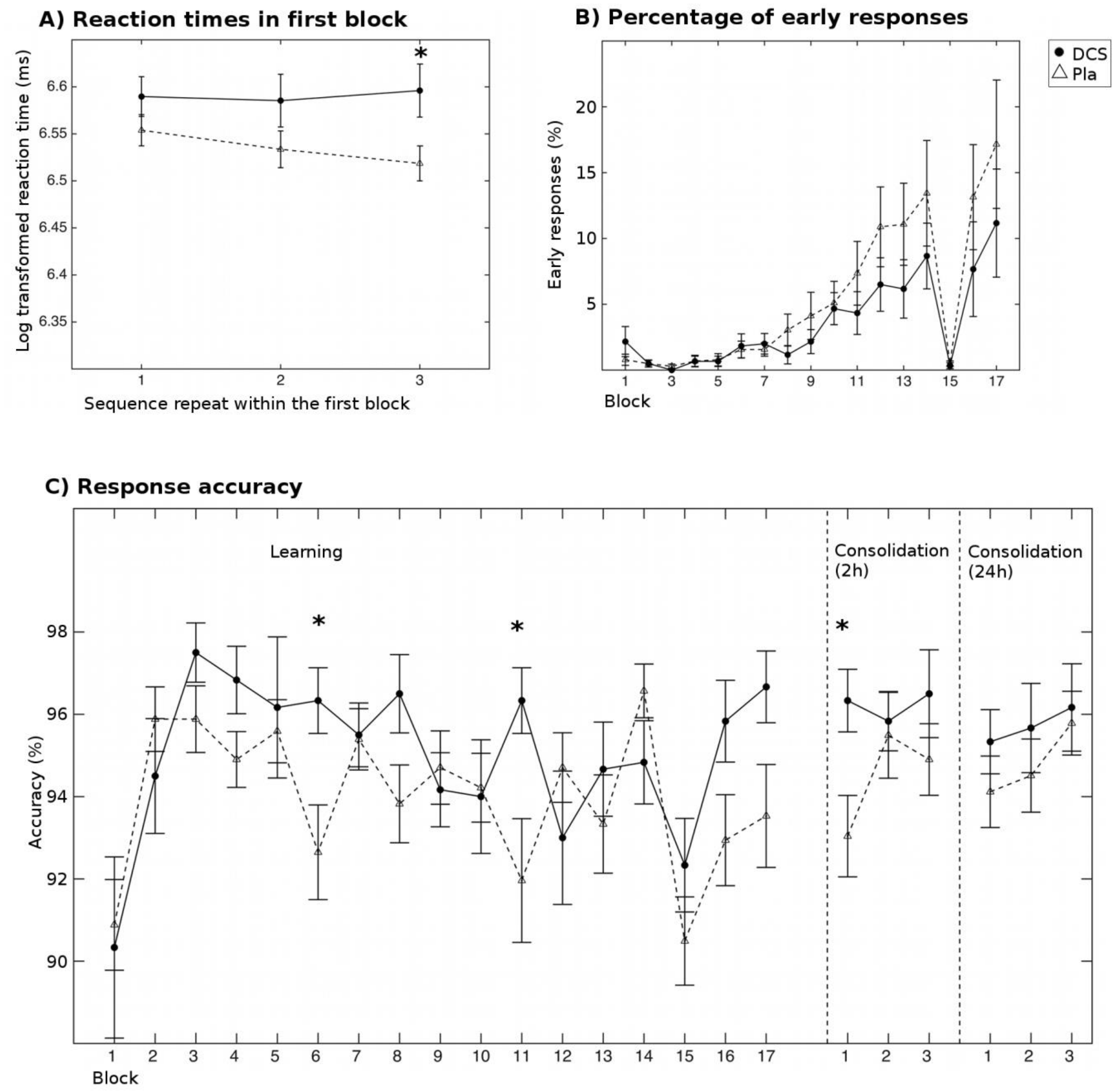

Figure S2. Additional behavioral results. A) Log-transformed RTs for the first three sequence repeats within the first learning block (block 2). B) Percentage of early responses (i.e. within 200ms of trial onset). C) Accuracy (%) for the initial learning and the two consolidation phases. Error bars show the standard error of the mean, *p<0.055

Supplementary Tables

	Pla (n=34)	DCS (n=20)	p
**Age**	22.2±0.5	22.3±0.7	0.86
**Gender, F:M**	17:17	11:9	0.78
**BDI**	1.7±0.4	0.9±0.4	0.25
**Education years**	16.6±2.1	15.9±2.3	0.71
**Trait anxiety**	30.7±1.3	30.5±1.3	0.94
**Weight**	66.2±1.8	63.7±9.3	0.38
**Neuroticism**	5.1±0.7	5.6±0.9	0.73
**ACS**	61.4±0.9	59.7±1.6	0.33
**BIS**	15.9±3.5	16.7±3.5	0.45
**BAS**	24.8±1.0	23.6±1.1	0.43
**ASI**	16.2±2.4	10.8±1.4	0.11

Table S1. Socio-demographic and questionnaire measurements for the placebo (pla) and the d-cycloserine (DCS) groups collected at baseline. Values are means and standard errors; p-values are for two-sided between-subject t-tests. Abbreviations and references for the questionnaires (see also methods): BDI [Beck Depression Inventory, Beck et al. (1996)], Trait anxiety (Spielberger and Gorsuch, 1983), Neuroticism (Eysenck and Eysenck, 1975), ACS [Attention Control Scale, Derryberry and Reed (2002)], BIS /BAS [Behavioral Inhibition/ Behavioral Activation Scale, Carver and White (1994)], ASI [Anxiety Sensitivity Index, Taylor and Cox (1998)].

VAS item	Pla		DCS		p		
	before	after	before	after	before	after	diff.
**Anxious**	7.4±1.4	3.7±0.6	6.7±1.5	5.7±1.8	0.73	0.21	0.23
**Sleepy**	27.7±3.4	20.9±3.6	24.1±3.3	13.7±2.5	0.49	0.16	0.44
**Flushed**	8.5±1.7	3.4±0.7	7.1±1.9	3.7±0.7	0.59	0.76	0.48
**Tearful**	3.0±0.7	2.7±0.7	3.1±6.7	2.8±0.5	0.96	0.94	0.98
**Nauseous**	3.1±0.7	2.9±0.8	2.9±0.7	3.5±0.6	0.84	0.63	0.32
**Hopeless**	3.2±0.6	2.4±0.4	4.1±1.5	2.7±0.5	0.53	0.67	0.64
**Tremor**	3.3±0.7	3.5±0.9	3.7±1.0	3.1±0.6	0.72	0.75	0.41
**Sad**	4.5±0.8	2.7±0.4	4.5±1.3	2.8±0.5	0.98	0.8	0.87
**Dizzy**	2.7±0.6	3.3±0.9	3.0±0.7	5.4±2.0	0.77	0.29	0.26
**Depressed**	2.6±0.4	2.3±0.4	3.1±0.8	2.5±0.5	0.53	0.79	0.52
**Tachycardia**	4.7±1.2	3.2±0.9	5.7±1.5	3.6±0.7	0.6	0.8	0.61
**Alert**	49.8±4.3	46.5±4.6	57.8±5.0	52.3±5.4	0.24	0.43	0.56

Table S2. Visual analogue scale measurements for the placebo (pla) and d-cycloserine (DCS) groups. Data was acquired before the administration of DCS or placebo and after. Values are means and standard errors as indicated by participants by placing a tick on a line of length 100mm. P-values are for two-sided between-subject t-tests of the scores before or after DCS/ placebo administration or the difference (diff., i.e. before minus after).

Supplementary methods and results
Study inclusion criteria
Inclusion criteria for participants were: no history of neurological or axis 1 psychiatric disorder as assessed using the Structured Clinical Interview for DSM-IV Axis I Disorders SCID-CV (First, 1996), no first-degree family member with a history of a severe psychiatric disease, passing a basic medical screening and examination for any type of medical illness relevant to the study, no current or recent (6 weeks) CNS-active medication or medication with cycloserine, ethionamide or isoniazid, a body mass index (BMI) between 18-30 kg/m^2, non- or light-smoking (<5 cigarettes a day), fluent English skills. Female participants were neither pregnant nor breast-feeding. This experiment was collected as part of a larger study in which a third drug (hydrocortisone) was also used (data not reported here).

Assessing initial learning and maximum improvement separately
Our results in the main report showed that learning across the whole experiment was not affected by DCS. To test whether there were maybe more subtle learning differences, we tested whether DCS enhanced either the initial speed of learning or the maximum improvement in performance. To test for effects on the initial speed of learning, we performed an ANOVA over the first four learning blocks (i.e. blocks 2 to 5). To assess the maximum improvement in performance, we compared the difference score between the last and the first learning block between the two groups using an independent-sample two-tailed t-test.

In terms of the early speed of learning, we again found that participants showed learning (ANOVA, effect of block: $F(2.2,115.9)=23.5$, $p<0.001$). This early learning appeared to differ between the groups, with the DCS group showing faster learning (ANOVA, block x group: $F(2.2,115.9)=3.4$, $p=0.032$). However, a closer look at the data (figure S2A) revealed that this result was not actually due to DCS enhancing the learning speed. Within each block, there were three repeats of the 10-element sequence; an analysis of these first three repeats within the first learning block (figure S2A) revealed that, in fact, the placebo group already showed more learning within that first learning block, i.e. block 2 ($t(52)=-2.1$, $p=0.043$ for the difference between the third and the first sequence repeat within the first block). This suggested that the steeper difference in RT in the DCS compared to the placebo group when comparing the early learning blocks (figure 1A) should not be interpreted as faster learning in the DCS group. Instead, the data suggests if anything that DCS may have slowed down very early learning within the first learning block.

Next, we analyzed whether DCS affected the overall amount learnt, i.e. the difference between the RT of the last and the first learning block. We did not find a difference between the two groups ($t(52)=-0.6$, $p=0.52$). This result did not change when the initial learning performance (block 2) was included as a covariate ($F(1,53)=0.026$, $p=0.87$)

Anticipatory responses in early learning
In the analyses above and in the main paper, we included RTs that occurred before the appearance of the cue (see Methods in main paper). The reason for this was that we noted that with learning participants started to respond in anticipation rather than in response to the cues appearing. Figure S2B shows the percentage of anticipator RTs, i.e. RTs faster than 200ms after cue onset. Both groups started showing more anticipatory responses as they progressed and learned in the task. The cut-off of 200ms was chosen based on RTs in the ‘random block’ (block 15). Here, we found that the mean

across participants of the minimum RT was 259±10ms (256ms in the placebo and 254ms in the DCS group). In this ‘random block’ all button presses could only be in response to the cue as there was no underlying sequence which participants could have used to predict the next button press. Therefore, the minimum RT in this block should be close to the fastest reactive speed participants could also have in the other blocks. In the last learning block, the block with the largest percentage of anticipatory responses, the average RT of these was 58.7±15.6ms. The groups did not differ in their percentage of early responses in any blocks of the experiment (all $p>0.18$).

Supplementary References

Beck AT, Steer RA, Brown GK (1996) Beck depression inventory-II. San Antonio.

Carver CS, White TL (1994) Behavioral inhibition, behavioral activation, and affective responses to impending reward and punishment: the BIS/BAS scales. Journal of personality and social psychology 67:319.

Derryberry D, Reed MA (2002) Anxiety-related attentional biases and their regulation by attentional control. Journal of Abnormal Psychology 111:225-236.

Eysenck HJ, Eysenck SBG (1975) Eysenck Personality Questionnaire (Junior & Adult): EdITS/Educational and Industrial Testing Service.

First MB (1996) User's guide for the structured clinical interview for DSM-IV axis I disorders: SCID-I: Biometrics Research Department, New York State Psychiatric Institute.

Spielberger CD, Gorsuch RL (1983) State-trait anxiety inventory for adults: Manual, instrument, and scoring guide: Mind Garden, Incorporated.

Taylor S, Cox BJ (1998) An expanded anxiety sensitivity index: evidence for a hierarchic structure in a clinical sample. Journal of anxiety disorders 12:463-483.
